# New insights in ferroptosis: Potential therapeutic targets for the treatment of ischemic stroke

**DOI:** 10.3389/fphar.2022.1020918

**Published:** 2022-11-08

**Authors:** Ziqing Wei, Yi Xie, Mingze Wei, Huijuan Zhao, Kaidi Ren, Qi Feng, Yuming Xu

**Affiliations:** ^1^ Department of Neurology, The First Affiliated Hospital of Zhengzhou University, Zhengzhou, China; ^2^ Henan Key Laboratory of Cerebrovascular Diseases, The First Affiliated Hospital of Zhengzhou University, Zhengzhou, China; ^3^ Clinical Systems Biology Laboratories, The First Affiliated Hospital of Zhengzhou University, Zhengzhou, China; ^4^ The Second Clinical Medical College, Harbin Medical University, Harbin, China; ^5^ Henan International Joint Laboratory of Thrombosis and Hemostasis, Basic Medical College, Henan University of Science and Technology, Luoyang, China; ^6^ Department of Pharmacy, The First Affiliated Hospital of Zhengzhou University, Zhengzhou, China; ^7^ Henan Key Laboratory of Precision Clinical Pharmacy, Zhengzhou, China; ^8^ Henan Engineering Research Center for Application & Translation of Precision Clinical Pharmacy, Zhengzhou University, Zhengzhou, China; ^9^ Research Institute of Nephrology, The First Affiliated Hospital of Zhengzhou University, Zhengzhou, China; ^10^ Department of Integrated Traditional and Western Nephrology, The First Affiliated Hospital of Zhengzhou University, Zhengzhou, China; ^11^ Henan Province Research Center for Kidney Disease, The First Affiliated Hospital of Zhengzhou University, Zhengzhou, China

**Keywords:** stroke, neurovascular unit (NVU), ferroptosis, inhibitors, therapeutic target

## Abstract

Stroke is a common disease in clinical practice, which seriously endangers people’s physical and mental health. The neurovascular unit (NVU) plays a key role in the occurrence and development of ischemic stroke. Different from other classical types of cell death such as apoptosis, necrosis, autophagy, and pyroptosis, ferroptosis is an iron-dependent lipid peroxidation-driven new form of cell death. Interestingly, the function of NVU and stroke development can be regulated by activating or inhibiting ferroptosis. This review systematically describes the NVU in ischemic stroke, provides a comprehensive overview of the regulatory mechanisms and key regulators of ferroptosis, and uncovers the role of ferroptosis in the NVU and the progression of ischemic stroke. We further discuss the latest progress in the intervention of ferroptosis as a therapeutic target for ischemic stroke and summarize the research progress and regulatory mechanism of ferroptosis inhibitors on stroke. In conclusion, ferroptosis, as a new form of cell death, plays a key role in ischemic stroke and is expected to become a new therapeutic target for this disease.

## Introduction

Stroke is a common disease in clinical practice, which seriously endangers people’s health and is mainly divided into two subtypes, including ischemic and hemorrhagic stroke (Peisker et al., 2017). Current evidence suggests that ischemic stroke accounts for approximately 85% of the morbidity of stroke (Benjamin et al., 2018), mainly due to cerebral blood circulation disorder, localized brain tissue necrosis or softening caused by ischemia and hypoxia, leading to corresponding nervous system function defects (Sacco et al., 2013). Ischemic stroke is also a serious disease with high mortality, and the resulting severe cognitive and motor impairments can significantly burden families and society (Owolabi et al., 2015; Donkor, 2018). The post-ischemic brain is characterized by the accumulation of amyloid plaques and neurofibrillary tangles, followed by the development of dementia (Yang et al., 2019). Therefore, ischemic stroke increases the neurological deficits in dementia patients (Owolabi et al., 2015).

It is widely thought that the neurovascular unit (NVU) plays a crucial role in the occurrence and development of ischemic stroke (Iadecola, 2017), as well as in the remodeling of blood vessels and nerves after stroke (Leigh et al., 2018). The past decade has witnessed significant inroads in pathological research on ischemic stroke with the discovery of a new form of cell death in the NVU of ischemic stroke, namely ferroptosis (Doll et al., 2017; Magtanong and Dixon, 2018; Zhou et al., 2021).

In recent years, ferroptosis has become a research hotspot (Dixon et al., 2012). During ferroptosis, a high abundance of unsaturated fatty acids on the cell membrane undergo lipid peroxidation under ferrous iron or ester oxygenase, thereby inducing cell death (Yang et al., 2016). The occurrence and execution of ferroptosis depend on the interaction of amino acid, lipid and iron metabolism (Ursini and Maiorino, 2020), and its sensitivity is also regulated by several key pathways and processes (Chen et al., 2021a). Ferroptosis is associated with various diseases such as Parkinson’s disease (Mahoney-Sanchez et al., 2021), tumor (Kim et al., 2016), and renal failure (Adedoyin et al., 2018), and the development of these diseases can be intervened by activating or inhibiting ferroptosis.

In ischemic stroke, pathological changes are closely related to ferroptosis, such as iron metabolism disorder, lipid peroxidation, and increased ROS (Hu et al., 2019; Ren et al., 2020). An increasing body of evidence from recently published studies substantiates the correlation between ferroptosis and stroke (Hu et al., 2019; Ren et al., 2020). This review provides a comprehensive overview of the NVU of ischemic stroke and the role of ferroptosis in ischemic stroke, providing new insights into the application of ferroptosis in treating ischemic stroke.

## NVU in ischemic stroke

**FIGURE 1:**
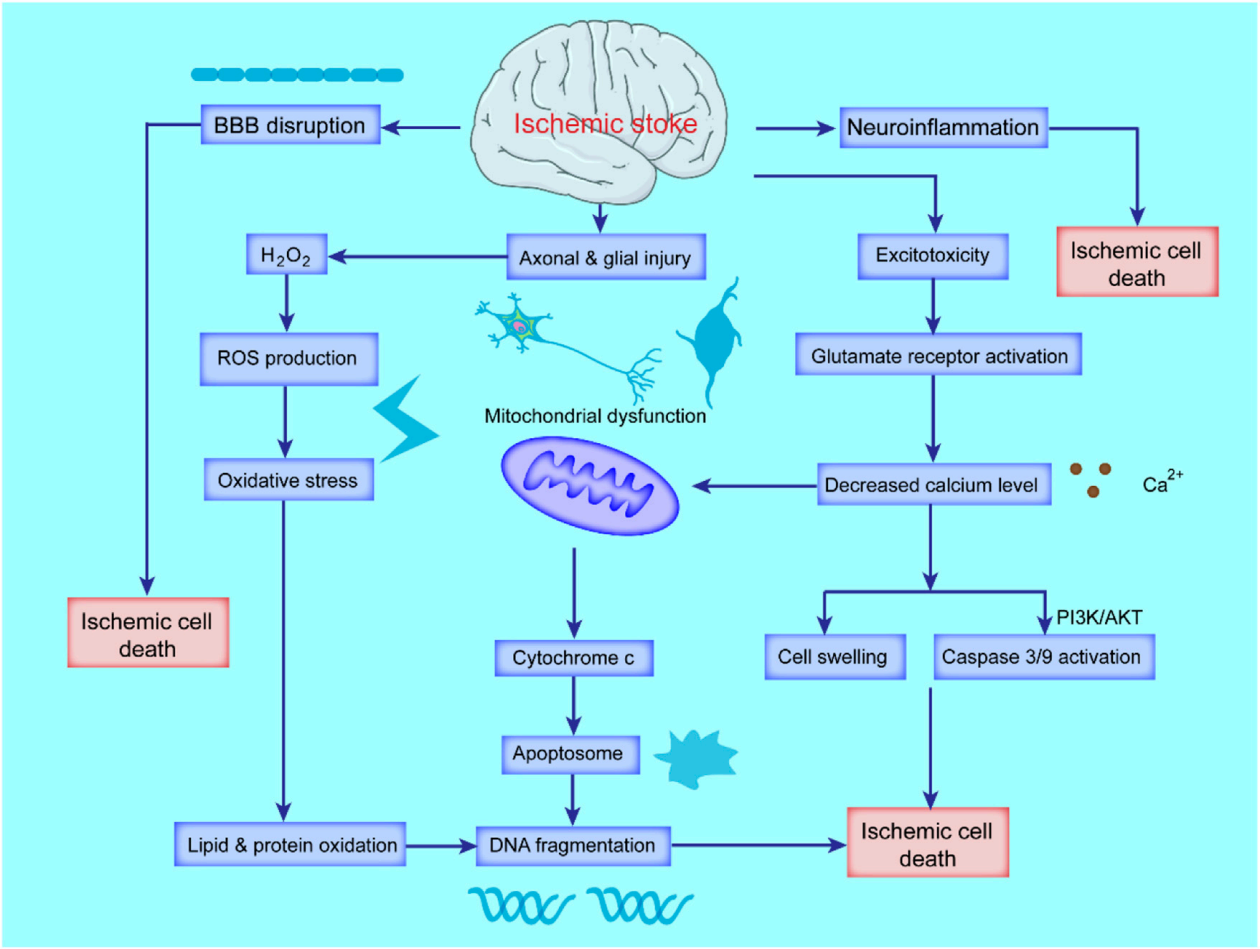
Schematic diagram of the pathological mechanism of ischemic stroke. Ischemic stroke triggers cascades of complex events that cause oxidative stress and excitotoxicity due to the accumulation of ROS and calcium (Ca^2+^), blood-brain-barrier (BBB) breakdown and activated inflammatory responses. Excessive ROS and Ca^2+^ lead to mitochondrial dysfunction and activation of apoptotic factors, ultimately leading to apoptosis and necrotic cell death.

Although significant progress has been made in better understanding the mechanism of neuron injury and repair after ischemia (Figure 1), there is still a lack of effective treatment for this disease (Chan, 1996; Hu et al., 2017; Yang et al., 2017). In the past, research on cerebral ischemic injury was mostly limited to neurons (Yang et al., 2019) or different cell groups and structures in the brain (Yang et al., 2017; Qin et al., 2019), ignoring the integrity of brain function and the interaction between different structures. Recently, the concept of the neurovascular unit as a new protective target for ischemic brain injury has been proposed (Cai et al., 2017; Zhao et al., 2020a).

The neurovascular unit is mainly composed of neurons, glial cells (including astrocytes, microglia, oligodendrocytes), and the blood-brain barrier (BBB, including vascular endothelial cells, astrocytic end-foot processes, basal lamina and pericytes) to maintain homeostasis of the central system (Pardridge, 1991), and extracellular matrix that maintains the integrity of the brain tissue environment (Lo and Rosenberg, 2009; Iadecola, 2017). The NVU maintains the normal physiological function of neurons and the repair of damaged neurons, which emphasizes the importance of the interconnection and mutual influence between neurons, glial cells and cerebrovascular (Stamatovic et al., 2008; Lo and Rosenberg, 2009) and provides the foothold for further study of neuron injury and protection mechanism. Overall, the NVU plays a key role in the clinical treatment of ischemic stroke and is increasingly valued by researchers and clinicians (Iadecola, 2017).

### Neurons

It is well-established that neurons are most vulnerable to cerebral ischemia-reperfusion injury (CIRI) (Lo and Rosenberg, 2009) and are continuously affected by several pathological reactions such as inflammation, excitatory amino acid toxicity, and oxidative stress after CIRI (Rohnert et al., 2012). Among them, excitatory amino acids include glutamate and aspartate (Brann and Mahesh, 1994), glutamate is the main excitatory neurotransmitter in the mammalian central nervous system, which can have long-term effects on the structure and function of neurons, and glutamate-mediated excitatory signal transduction can affect mammalian brain functions, including cognition, memory and learning. The release of a large number of excitatory amino acids will activate plenty of ion channels and lead to persistent intracellular Ca^2+^ level increase, cell damage and death, which is called excitatory amino acid toxicity (Gillessen et al., 2002). While, this excitotoxicity caused by excitatory amino acids is one of the earliest and widely recognized molecular mechanisms of CIRI (Lai et al., 2014). When the brain is in a state of ischemia and hypoxia, the release of excitatory neurotransmitters is increased and reuptake is impaired due to metabolic disorders, and eventually the level of excitatory neurotransmitters in the ischemic region increases rapidly that leads to aberrant activation of many Ca^2+^-dependent pathways and initiation of apoptosis, necroptosis and autophagy processes in the brain (Shen et al., 2022). Current evidence suggests that neuronal death accounts for the poor prognosis in ischemic stroke (Chen et al., 2020). Indeed, assessing the severity of an ischemic stroke and the cause of death depends largely on the number of neurons dying in the affected brain area (Lazarov and Hollands, 2016).

### Microglia

Microglia are innate immune cells in the brain, accounting for approximately 5–20% of glial cells (Benveniste, 1997). The main functions of microglia are to recognize pathogens, phagocytose necrotic or apoptotic cells, remove damaged neurons, tissue fragments, small and inactive synapses, infected small molecules and macromolecules, regulate T cell response, and induce inflammatory process (Nimmerjahn et al., 2005). In addition, microglia have extensive connections with other NVU cells (Thurgur and Pinteaux, 2019), which can regulate the microenvironmental homeostasis of NVU, and have positive significance for maintaining the barrier function of BBB (Abdullahi et al., 2018). When an ischemic stroke occurs, neurons activate microglia to differentiate into M1-and M2-phenotypes by releasing certain soluble factors and intracellular components (Akhmetzyanova et al., 2019; Qin et al., 2019). It is well-established that M1-type microglia have a pro-inflammatory and deleterious effect on the ischemic brain (Yu et al., 2022), while M2-type microglia can reduce the inflammatory response and exert neuroprotective effects (Varin and Gordon, 2009; Soehnlein and Lindbom, 2010).

In addition, microglia can affect the activity of neurons by releasing ATP and stimulating astrocytes to release glutamate to increase the excitatory postsynaptic potential (Barakat and Redzic, 2016; Illes et al., 2021). After cerebral ischemia, microglia release many inflammatory factors (Soehnlein and Lindbom, 2010), destroy the normal function of neurons and damage vascular endothelial cells, thereby destroying the BBB structure and aggravating brain edema (Yenari et al., 2010).

### Oligodendrocytes

Oligodendrocytes are the myelinating cells in the central nervous system (CNS) and originate from oligodendrocyte progenitor cells (OPCs). OPCs can differentiate into oligodendrocytes or astrocytes according to the environment (Hughes et al., 1988). Endothelial cells promote the proliferation of OPCs by releasing trophic factors such as brain-derived neurotrophic factor (BDNF) and basic fibroblast growth factor (bFGF) (Dugas et al., 2008) and release vascular endothelial growth factor A (VEGF-A) to promote oligodendrocyte migration (Guo et al., 2008; Arai and Lo, 2009). The main function of oligodendrocytes is to form an insulating myelin sheath wrapping the axons in the CNS, assist in the efficient transmission of bioelectrical signals, and maintain and protect the normal function of neurons (Baumann and Pham-Dinh, 2001; Kuhn et al., 2019). Abnormalities in oligodendrocytes not only lead to demyelinating lesions of the CNS but also cause neuronal damage, psychiatric diseases, and even brain tumors (Kuhn et al., 2019). Under ischemic conditions, the expression of Nogo-A in oligodendrocytes is upregulated (Kern et al., 2013), thereby inhibiting axonal remodeling, impairing neuronal function, and triggering the early breakdown of BBB by secreting matrix metalloproteinase-9 (Mandai et al., 1997; Dewar et al., 2003).

### Vascular endothelial cells

Vascular endothelial cells (VECs) constitute a monolayer of specialized cells strategically positioned between the vascular wall and the bloodstream (Kruger-Genge et al., 2019). Ischemic stroke results from a combination of factors such as platelet adhesion and aggregation and related release reactions (Del Zoppo, 1998), fibrin protease activation, and fibrin formation after vascular endothelial injury (Zhou et al., 2020). Under normal conditions, certain active factors released by VECs play a protective role in regulating vascular tension, coagulation, fibrinolysis, and maintaining normal blood pressure and hemodynamics (Sandoo et al., 2010). Once the vascular endothelium is damaged, the exposed subendothelial layer can cause platelet adhesion and aggregation, leading to thrombosis (Chen and Lopez, 2005). The stimulated VECs can also release tissue factors to promote the extrinsic coagulation process involving coagulation factor XII and accelerate thrombosis (Lopes-Bezerra and Filler, 2003). Notably, prolonged ischemia-hypoxia and ischemia-reperfusion (I/R) can damage VECs (Zhou et al., 2020). In addition, ischemia and hypoxia can induce the expression of VEGF (Ramakrishnan et al., 2014), which can promote the proliferation of VECs and participate in angiogenesis, thereby suppressing ischemic stroke and playing a neuroprotective role (Lopes-Bezerra and Filler, 2003; Zhou et al., 2020).

### Astrocytes

In glial cells, astrocytes perform multiple homeostatic functions to maintain the survival and stability of the NVU (Becerra-Calixto and Cardona-Gomez, 2017), exerting neuroprotective, angiogenic, immunomodulatory, neurogenic, and antioxidant effects with the ability to modulate synaptic function (Daneman and Prat, 2015; Becerra-Calixto and Cardona-Gomez, 2017). Under physiological conditions, astrocytes release various neurotrophic factors, which can repair damage to neurons and VECs (Ye et al., 2018). During cerebral ischemia, the energy supply of brain cells is insufficient, resulting in the dysregulation of intracellular calcium and sodium pumps (Peng et al., 2019). Because of the extensive gap junctions and hemichannels in astrocytes, the gap junctions are destroyed, and Ca^2+^ and toxic substances are rapidly transmitted, causing astrocytes to release a large amount of glutamate to aggravate the excitatory amino acid toxicity (Lim et al., 2021). On the other hand, astrocytes are overactivated in the acute phase of ischemia, eventually forming a glial scar that hinders the repair of neurons (Sofroniew, 2009).

### Pericytes

Brain pericytes are located in the center of the NVU and respond by receiving, integrating and processing signals from neighboring cells (Hamilton et al., 2010; Armulik et al., 2011; Winkler et al., 2011). They are critical in maintaining the normal function of the CNS and are involved in the formation and maintenance of BBB, cerebral blood flow (CBF) regulation, immunoregulation, angiogenesis, and stability. Overwhelming evidence substantiates that the dysfunction and loss of pericytes play a key role in the pathogenesis of various cerebrovascular diseases (Winkler et al., 2011). It has been shown that after ischemia, pericytes begin to detach from the cerebral microvessels (Zhou et al., 2022), which causes the destruction of tight junctions between cells, resulting in the destruction and leakage of the BBB (Bergers and Song, 2005). The platelet-derived growth factor receptor β (PDGFRβ) on pericytes is upregulated after cerebral ischemia and can combine with PDGFβ secreted by VECs to promote the recruitment and migration of pericytes for neovascularization to promote maturation (Makihara et al., 2015; Hutter-Schmid and Humpel, 2016). It has been shown that pericytes begin to secrete VEGF within 24 h after ischemic stroke, which promotes angiogenesis in the peri-infarct area by activating VEGFR in endothelial cells (Shibuya, 2011; Yang et al., 2017; Zhou et al., 2022). Under ischemic and hypoxic conditions, pericytes can exhibit pluripotent stem cell properties and participate in immune responses (Davidoff, 2019; Fernandez-Morales et al., 2019). A recent study found that TNF-α could promote the release of IL-6 from pericytes, which contributed to the activation of microglia (Matsumoto et al., 2018a).

This NVU theory emphasizes the important connection between neurons, glial cells and microvessels after cerebral ischemia, corroborating that all components of NVU are involved in the pathological process of cerebral ischemia injury (Stamatovic et al., 2008; Mcconnell et al., 2017). This connection is realized through cell-cell and cell-matrix interaction. There are many chemicals involved in the regulation of BBB permeability in different cell types of NVU. These regulatory chemicals fall into different categories, such as proinflammatory cytokines (e.g., TNF-α, IL-1, IL-6), neurotransmitters (e.g., NO), ROS and other substances (Table 1). Comprehensive treatment of NVU can effectively combat ischemic stroke. Therefore, NVU provides a theoretical basis for the current research and treatment of neurological diseases, and targeting NVU from a global perspective may bring new opportunities for treating ischemic stroke.

**TABLE 1:** The functions and regulators of NVU.

NVU components	Functions	Regulators	References
Neurons	Critical in the regulation of BBB function, innervate endothelial cells and their associated astrocytes. Maintain the homeostasis of the brain microenvironment, provide nutritional support for the brain*etc.*	Mcl-1/Bcl-2, OXR1, P53/Caspase-3, TRAF3, ADIPOR2*etc.*	Awooda et al. (Awooda et al., 2015)
			Anilkumar et al. (Anilkumar et al., 2020)
			Cregan et al. (Cregan et al., 1999)
Astrocytes	As a part of the blood-brain barrier, can connect capillaries and neurons, participate in the nutritional support of neurons and the regulation of electrophysiological activities, and can secrete a large number of neurotrophic factors and growth factors to maintain the stability of the microenvironment and repair after injury	AQP-4, TLR4, TGF-β, ADIPOR2, Nrf2, ApoE, MCSF, IL-6, MCP-1, MMP-9, GFAP, GLT-1, GLAST, PARs*etc.*	Becerra-Calixto et al. (Becerra-Calixto and Cardona-Gomez, 2017)
			Hiroko (Ikeshima-Kataoka, 2016)
			Cekanaviciute et al. (Cekanaviciute and Buckwalter, 2016)
Microglia	As an innate immune effector cell, microglia is necessary for the normal development of the nervous system	TLR, MHC-II, CD16/32, BDNF, GDNF, VEGF, BMP-7, TGF-β, CSF-1, TNF-α, TNF-β, IGF-1, NADPH oxidase, IL-1, IL-4, IL-5, IL-6, IL-8, IL-10, pro-MMP-9, NO, ROS*etc.*	Kim et al. (Kim and De Vellis, 2005)
			Hamel (Hamel, 2006)
			Colonna et al. (Colonna and Butovsky, 2017)
			Kang et al. (Kang et al., 2020)
Oligodendrocytes	Their main function is to wrap axons in the central nervous system, form an insulating myelin structure, assist in the efficient transmission of bioelectrical signals, maintain and protect the normal function of neurons	Nogo-A, CNTF, IGF-1, NT-3, PDGF*etc.*	Nave et al. (Nave and Werner, 2014)
			Plemel et al. (Plemel et al., 2014)
Vascular endothelia cells (VECs)	As the physical barrier of the BBB, VECs are formed by preventing cells and molecules from passively entering the brain through tight junctions between cells	NF-κB, NO, Prostacyclin, EDHF, Eicosanoids, TIMP-2, VCAM-1, ICAM-1, P-selectin, MMPs*etc.*	Onat et al. (Onat et al., 2011)
			Henke et al. (Henke et al., 2007)
			Kathrina et al. (Marcelo et al., 2013)
Pericytes	Vital in the formation and maintenance of BBB integrity, angiogenesis, and removal of toxic substances	Angiopoientin-1, MIF, Occludin, SIPT1, MRP*etc.*	Rustenhoven et al. (Rustenhoven et al., 2017)
			Hori et al. (Hori et al., 2004)
Extracellular matrix (ECM)	Mediating cell differentiation, proliferation, adhesion, morphogenesis and phenotype	Collagen, Undulin, Tenascin, Fibronectin, Dermatan sulfate, Decorin*etc.*	Bonnans et al. (Bonnans et al., 2014)
			Zhang et al. (Zhang et al., 2021a)
Basal lamina	Located on the outside of the lumen of the cerebral microvascular endothelium, it is composed of extracellular matrix proteins secreted by VECs, pericytes, and astrocytes, and is involved in the regulation of vascular integrity	Collagen, Laminin, Fibronectin, Elastin, Proteoglycans, Merosin, Dystroglycan, Nidogen, Growth factors, MMPs*etc.*	Hoshi et al. (Hoshi and Ushiki, 2004)
			Nguyen et al. (Nguyen et al., 2021)

## Mechanisms and key regulators of ferroptosis

Programmed cell death plays an important role in homeostasis and disease development. Among them, ferroptosis is a newly discovered form first proposed by Stockwell et al., in 2012 (Dixon et al., 2012). Ferroptosis is significantly different from other types of cell death such as apoptosis, necrosis, autophagy, and pyroptosis at the morphological, biochemical, and genetic levels (Dixon et al., 2012). The morphological features of ferroptosis are mainly manifested in mitochondria, including reduced volume, increased membrane density, and reduced numbers or absence (Chen et al., 2021a; Jiang et al., 2021). Regarding biochemical characteristics, ferroptosis manifests as glutathione depletion, glutathione peroxidase 4 (GPX4) inactivation, and lipid peroxide accumulation (Chen et al., 2021a; Jiang et al., 2021).

**FIGURE 2:**
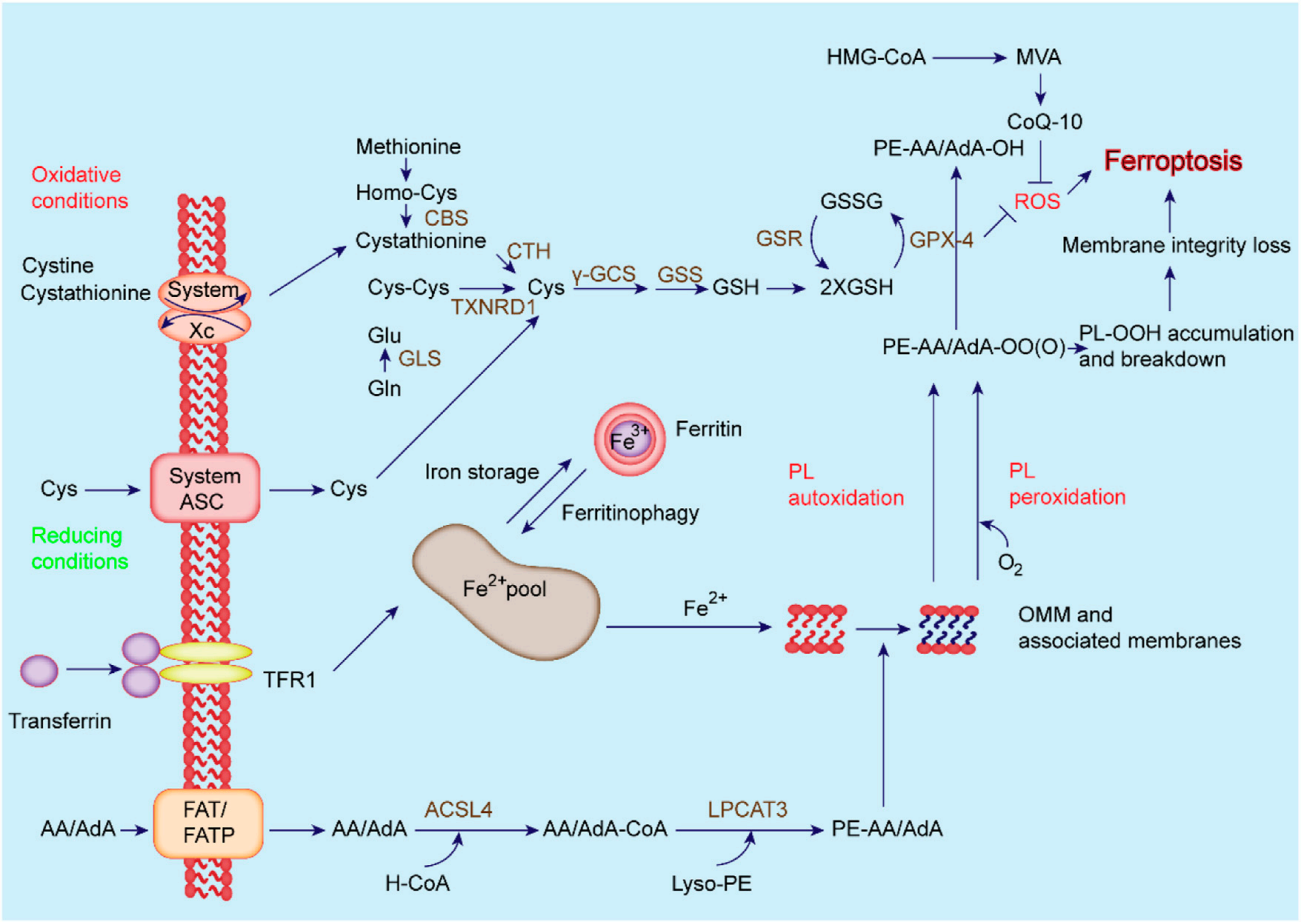
Regulatory mechanisms of ferroptosis. The primary metabolism involved in ferroptosis can be roughly divided into three categories: iron metabolism, System Xc^−^/GSH/GPX4 pathway, and lipid peroxidation. Besides, the FSP1-CoQ_10_-NAD(P)H pathway, which exists as an independent parallel system with GPX4 and GSH, inhibits phospholipid peroxidation and ferroptosis.

It has been confirmed that ferroptosis is closely related to neurodegenerative diseases, tumors, cardiovascular and cerebrovascular diseases, and acute kidney injury (AKI) (Ward et al., 2014; Fang et al., 2019; Bao et al., 2021), and its inhibitors can effectively delay disease progression and improve clinical symptoms (Angeli et al., 2017). However, the regulatory mechanism of ferroptosis has not yet been fully elucidated. With significant progress achieved in the study of ferroptosis, various regulatory factors and mechanisms have been discovered, suggesting that it is mainly related to iron metabolism disorder, amino acid antioxidant system imbalance, and lipid peroxide accumulation **(** Figure 2) (Dixon et al., 2012; Chen et al., 2021a). When iron metabolism disorder causes the increase of intracellular free iron, iron catalyzes the production of ROS through the Fenton reaction, and ROS further promotes lipid peroxidation, causing the accumulation of lipid peroxides and inducing ferroptosis (Dixon et al., 2012; Stockwell et al., 2017; Chen et al., 2021a). Indeed, an in-depth study and elucidation of the pathophysiological mechanism of ferroptosis can provide new ideas and treatment methods for ferroptosis-related diseases.

### Iron metabolism

Iron metabolism disorders, especially iron overload, are key to ferroptosis (Dixon et al., 2012). Fe^3+^ in the blood circulation is combined with transferrin and transported to the cell through transferrin receptor 1 (TFR1) on the cell membrane surface (Beguin et al., 2014). Then Fe^3+^ is reduced to Fe^2+^ and released into the labile iron pool (LIP) in the cytoplasm (Kakhlon and Cabantchik, 2002), while excess iron is stored in ferritin (Jacobs et al., 1972). During this process, nuclear receptor coactivator 4 (NCOA4) acts as an adaptor protein to mediate the targeted transport of ferritin to lysosomes for autophagic degradation, thereby releasing free Fe^2+^, a process called ferritinophagy, mainly responsible for iron release and recovery (Mancias et al., 2014). Part of Fe^2+^ is transported out of cells through ferroportin1 (FPN1) on the cell membrane to ensure that the intracellular iron concentration is not excessively high under physiological conditions (Zhang et al., 2011; Zhang et al., 2012). Current evidence suggests that iron metabolism disorders can increase intracellular LIP and cause an increase in intracellular free iron (Ravingerova et al., 2020).

Due to the instability and high reactivity of Fe^2+^, hydroxyl radicals can be generated through the Fenton reaction (Thomas et al., 2009), which can directly react with polyunsaturated fatty acids (PUFAs) in the plasma membrane to generate a large amount of lipid ROS (Magtanong et al., 2016), and further promote lipid peroxidation and peroxide accumulation, inducing cell ferroptosis (Stockwell et al., 2017).

### System Xc^−^

The cystine-glutamate antiporter System Xc^−^ is widely distributed in the phospholipid bilayer of biological cells (Bannai, 1986; Sasaki et al., 2002; Bridges et al., 2012). It is a heterodimer composed of light chain solute carrier family seven member 11 (SLC7A11) and heavy chain solute carrier family three member 2 (SLC3A2) (Sato et al., 1999; Broer and Wagner, 2002; Verrey et al., 2004). The System Xc^−^ can export intracellular glutamate to the extracellular space while importing cystine into the cytoplasm, where cystine is reduced to cysteine, which is involved in glutathione (GSH) synthesis (Seib et al., 2011). Glutathione peroxidase (GPX), whose active center is selenocysteine, catalyzes the conversion of reduced GSH to oxidized glutathione (GSSG), converting toxic peroxides into hydroxyl compounds to protect cell membranes from oxidative stress damage (Guan et al., 2017).

GPX4 is the only enzyme found in the GPX family that can reduce peroxides in lipid membranes, and its antioxidant effect is significantly higher than other family members (Margis et al., 2008). In particular, GPX4 can degrade hydrogen peroxide and other small molecule peroxides induced by iron overload in cells, preventing ferroptosis caused by the accumulation of ROS (Borchert et al., 2018). The antioxidant activity of GPX4 depends on GSH, which acts as an electron donor and converts toxic lipid hydroperoxide into non-toxic lipid alcohol (L-OH) (Fei et al., 2020). When System Xc^−^ is blocked, glutamate and cystine cannot be exchanged, resulting in the accumulation of intracellular glutamate, decreased GSH synthesis and GPX4 activity, thereby increasing ROS in lipids and inducing cell ferroptosis (Forcina and Dixon, 2019).

### Lipid peroxidation

Lipid peroxidation refers to the loss of hydrogen atoms of lipids under the action of free radicals or lipid peroxidase, resulting in the oxidation, fragmentation and shortening of lipid carbon chains and the production of lipid free radicals, lipid hydroperoxides (LOOH) and reactive aldehydes (such as malondialdehyde and 4-hydroxynonenal) and other cytotoxic substances, eventually cause lipid oxidative degradation reactions that damage cells (Ayala et al., 2014). ROS are a group of molecules with partially reduced oxygen, including peroxides, superoxides, singlet oxygen, free radicals, *etc.*, which cause cell death by damaging DNA, RNA and lipid molecules (Su et al., 2019; Villalpando-Rodriguez and Gibson, 2021). As a member of intracellular ROS, lipid peroxides are the ultimate executors of ferroptosis (Cheng et al., 2021). The deleterious effect of lipid peroxidation is mainly reflected in the oxidative degradation of two important biofilm components, including phosphatidylethanolamines (PEs) and PUFAs (Gaschler and Stockwell, 2017).

PUFA is the main component of phospholipids in cell and organelle membranes and is also an important substrate for the synthesis of PE (Stubbs and Smith, 1984). PUFA has a high affinity for free radicals, and the hydrogen atoms between its double bonds are easily oxidized by free radicals (Cunnane, 1994). The lipid peroxidation reaction of PUFA is roughly divided into two stages (Kanner et al., 1987; Girotti, 1998; Ayala et al., 2014). First, ROS acquire hydrogen atoms in PUFA to generate lipid radicals (Yin et al., 2011); subsequently, lipid radicals interact with oxygen molecules to generate lipid peroxyl radicals (LOO-) (Chamulitrat and Mason, 1989). LOO- can reportedly abstract hydrogen atoms from other PUFAs to form lipid radicals and lipid hydroperoxides (Chamulitrat and Mason, 1989; Girotti, 1998). Moreover, LOO- participates in the oxidation process of PUFAs, which ensures that the lipid peroxidation of PUFAs exhibits the characteristics of a cascade reaction (Yin et al., 2011).

However, the affinity between PE and free radicals is not high, and oxidation sites need to be formed under the action of two enzymes before lipid peroxidation occurs (Pratt et al., 2011). First, long-chain acyl-Coa synthetase-4 (ACSL4) utilizes arachidonic acid (AA) and adrenic acid (AdA) to synthesize arachidonoyl-CoA (AA-COA) and adrenoyl-CoA (AdA-COA) (Ma et al., 2021); then, AA/AdA-COA combines with PE to form PE-AA/AdA under the catalytic action of lysophosphatidylcholine acyltransferase 3 (LPCAT3) (Kagan et al., 2017).

PE-AA/AdA is easily oxidized to cytotoxic PE-AA/AdA-OOH by free radicals or Arachidonate 15-Lipoxygenase (ALOX15), which promotes ferroptosis (Kagan et al., 2017). LOX-mediated lipid hydroperoxide production has been suggested to be involved in ferroptosis (Shintoku et al., 2017). The accumulation of lipid peroxides, especially phospholipid peroxides, is a hallmark event of ferroptosis (Shintoku et al., 2017). High levels of ACSL4 and LPCAT3 have been detected in various tumors, such as renal and liver cancer cells (Luo et al., 2021). At present, the expression of these two enzymes has been used to assess the sensitivity of various tumor cells to ferroptosis (Yuan et al., 2016a; Doll et al., 2017; Kagan et al., 2017; Magtanong and Dixon, 2018).

### Nrf2

Nuclear factor-E2-related factor 2 (Nrf2) is a transcription factor with a leucine zipper structure, which plays a key anti-oxidation role (Jaiswal, 2004). The activity of Nrf2 is strictly regulated by Kelch-like ECH-associated protein 1 (Keap1) (Kobayashi and Yamamoto, 2006). Under normal conditions, Nrf2 binds to Keap1 and is inactivated with ubiquitination and degradation in the proteasome (Zhang et al., 2004). Once in a state of oxidative stress, Keap1 is degraded by autophagy to release Nrf2 (Kaspar et al., 2009). Free Nrf2 rapidly translocates to the nucleus, which binds to antioxidant response elements (AREs) in the promoter region to drive antioxidant gene expression, balance oxidative stress and maintain cellular redox homeostasis (Kwak et al., 2007).

It is well-established that Nrf2 can regulate a variety of antioxidant enzymes, such as superoxide dismutase (SOD), catalase (CAT), glutathione peroxidase (GPX), glutathione reductase (GR), NAD(P)H quinine oxidoreductase (NQO1) and so on (Dhakshinamoorthy et al., 2000; Zhu et al., 2005). Therefore, Nrf2 is considered an important regulator of ferroptosis and a therapeutic target for tumors and neurodegenerative diseases highly associated with oxidative stress (Abdalkader et al., 2018; Song and Long, 2020).

### P53

p53 has attracted much interest as a tumor suppressor molecule since its discovery (Harris, 1996). The p53 molecule can induce apoptosis and cell cycle arrest, exerting a strong tumor suppressor effect (Chen, 2016). In 2015, Jiang et al. linked p53 to ferroptosis for the first time (Jiang et al., 2015) and demonstrated that mutation of p53 can inhibit the activity of System Xc^−^, downregulate the expression of SLC7A11 (Jiang et al., 2015), and reduce the activity of GPX4, thereby promoting lipid peroxidation and inducing ferroptosis (Jiang et al., 2015). It is widely thought that p53 is at the core of a powerful signaling network; it regulates the sensitivity of cells to ferroptosis in different cell types and under different stress factors through several independent signaling pathways (Harris and Levine, 2005; Huang, 2021).

In addition to increasing sensitivity to ferroptosis, p53 appears to have an opposing effect (Liu et al., 2020a). When cells undergo cysteine deprivation, another signaling pathway is activated, with increased expression of wild-type p53 to induce p21 transcription or inhibit DPP4 binding to NOX1, ultimately inhibiting cell susceptibility to ferroptosis (Wang et al., 2012; Badgley et al., 2020). These two functions seem contradictory, but they are unified in cells. On the one hand, ferroptosis, as a form of regulated cell death, has physiological significance in the evolution of species, and p53 can achieve the purpose of removing abnormal cells and inhibiting tumorigenesis by increasing the sensitivity of cells to ferroptosis (Jiang et al., 2015; Liu et al., 2020a); on the other hand, when metabolic stress occurs, p53 can reduce the sensitivity of cells to ferroptosis by enhancing the ability to regulate ROS level to help normal cells survive the damage induced by various stress factors and promote cell survival (Tarangelo et al., 2018). Overwhelming evidence substantiates that cell types and p53 mutation sites may influence the mechanism of p53 in regulating cell ferroptosis (Wynford-Thomas and Blaydes, 1998; Wang et al., 2008; Ji et al., 2022), although the specific underlying mechanism warrants further study.

### FSP1

Previous studies suggested that GPX4 and free radical antioxidants regulate ferroptosis. Recently, Marcus Conrad and José Pedro Friedmann Angeli’s team used a clonal expression strategy to screen for genes that can inhibit ferroptosis caused by loss of GPX4 in human cancer cells and found that *flavoprotein apoptosis-inducing factor mitochondria-related 2 (AIFM2)* is an anti-ferroptosis gene and renamed AIFM2 to ferroptosis suppressor protein 1 (FSP1). FSP1, originally described as a pro-apoptotic gene, showed the ability to inhibit ferroptosis induced by *GPX4* knockout (Doll et al., 2019). In addition, the researchers found that the main mechanism of FSP1 in inhibiting ferroptosis is to reduce Coenzyme Q_10_ (CoQ_10_) with NAD(P)H, inhibit lipid peroxidation, and resist the occurrence of ferroptosis (Doll et al., 2019). The FSP1-CoQ_10_-NAD(P)H pathway exists as an independent parallel system, which, together with GPX4 and glutathione, inhibits phospholipid peroxidation and ferroptosis (Bersuker et al., 2019; Doll et al., 2019). Their anti-ferroptosis properties have been widely used in the study of anti-tumor therapy (Bersuker et al., 2019). Growing evidence suggests that when GPX4 is inactivated, FSP1 can continue to maintain tumor growth *in vivo*, while deletion of *GPX4* and *FSP1* can inhibit tumor growth (Doll et al., 2019). As a novel ferroptosis inhibitor, FSP1 provides a new direction for disease research.

### ACSL4

ACSL4 is a lipid metabolism enzyme required for lipid peroxidation and belongs to the ACSL family (Lopes-Marques et al., 2013). Current research suggests that PUFAs can be used as substrates for lipid peroxidation (Ayala et al., 2014), and their accumulation is a marker of ferroptosis; thus, the intracellular PUFAs content determines the development of ferroptosis and increased content can promote the progression of lipid peroxidation-induced ferroptosis (Das, 2019; Kuang et al., 2020). ACSL4 can activate free PUFAs and then complete the peroxidation process of membrane phospholipids with the participation of the other key enzymes, LPCAT3 and ALOX15 (Lee et al., 2021). Therefore, the ACSL4/LPCAT3/ALOX15 pathway can promote lipid peroxidation-induced ferroptosis (Lee et al., 2021). Zhang et al. showed that PKCβII, one of the isoforms of PKC (Steinberg, 2008), is an important lipid peroxidation sensor activated by lipid peroxidation during ferroptosis that can phosphorylate ACSL4 to amplify the effect of lipid peroxidation, eventually inducing ferroptosis (Zhang et al., 2022). This study also confirmed that the PKCβII-ACSL4 mechanism affects the efficacy of cancer immunotherapy by regulating ferroptosis (Zhang et al., 2022).

### Other regulatory mechanisms

Heat shock proteins (HSP) are a class of highly conserved molecular chaperones expressed in response to environmental stress and render cells resistant to different types of cell death (Feder and Hofmann, 1999). For example, HSPB1 can inhibit ferroptosis by reducing iron uptake (Sun et al., 2015); HSPA5 binds to and stabilizes GPX4, thereby indirectly avoiding the damage of lipid peroxidation in ferroptosis (Zhu et al., 2017); however, the HSP9 inhibitor CDDO can inhibit ferroptosis in tumor cells, suggesting that HSP90 may play a different role in ferroptosis (Qin et al., 2015).

Mitochondria have been reported to participate in the pathogenesis of ferroptosis (Gao et al., 2019). CDGSH iron-sulfur domain 1 (CISD1) is a class of mitochondrial iron-exporting proteins that inhibit ferroptosis by preventing the accumulation of iron and the production of ROS in mitochondria (Yuan et al., 2016b). In addition, voltage-dependent anion channels (VDACs) located in the mitochondrial outer membrane play an important regulatory role in ferroptosis (Lemasters, 2017). It has been shown that Erastin, a ferroptosis inducer (Shibata et al., 2019; Zhao et al., 2020b), can act on VDAC to promote the release of a large number of oxides, causing ROS-dependent mitochondrial dysfunction and bioenergy exhaustion to induce ferroptosis (Zhao et al., 2020b), while the reduction of VDAC expression can reduce the occurrence of Erastin-induced ferroptosis (Yang et al., 2020).

Epigenetics is a key factor in regulating gene expression, and more and more research results show that epigenetic regulation (e.g., DNA methylation, histone modification and miRNA) plays an important role in ferroptosis (Jaenisch and Bird, 2003; Wu et al., 2020). DNA methylation is the most widely studied epigenetic modification. Increased DNA methylation may lead to gene silencing, while decreased methylation activates gene expression (Newell-Price et al., 2000), and DNA methylation is closely related to iron metabolism and can control the expression of ferroptosis-related genes (e.g., *GPX4* and *SLC7A11*) to regulate ferroptosis (Zhao et al., 2022). Currently, DNA methylation is widely used as a diagnostic, predictive, and prognostic biomarker for multiple cancers (Huo et al., 2019). According to the histone modification studies, it was found that reducing histone 2A ubiquitination (H2Aub) on the *SLC7A11* promoter can downregulate SLC7A11 and prevent ferroptosis (Zhang et al., 2019a); the histone 2B monoubiquitination (H2Bub1) modification is significantly down-regulated during the induction of ferroptosis, and artificial inhibition of endogenous H2Bub1 can significantly increase the sensitivity of cells to the ferroptosis inducer Erastin (Wang et al., 2019); in addition, histone deacetylase (HDAC) can regulate iron metabolism by inhibiting *HAMP* gene expression (Sukiennicki et al., 2019). There are also many studies reporting that a large number of microRNAs (miRNAs) can regulate ROS metabolism and ferroptosis (Zhang et al., 2020a). Mitofusin (Mfn) is a key protein that maintains mitochondrial morphology, regulates cellular lipid metabolism, endoplasmic reticulum stress and ROS generation (Papanicolaou et al., 2011), and plays a potential role in ferroptosis (Wei et al., 2020). miR-195, miR-125a and miR-761 have all been reported to regulate the mitochondrial function and metabolism of breast cancer cells, pancreatic cancer cells and liver cancer cells by targeting *Mfn2*, respectively, and affect the growth of tumor cells (Guo et al., 2017; Pan et al., 2018; Purohit et al., 2019); miR-137 can negatively regulate ferroptosis by directly targeting glutamine transporter SLC1A5 in melanoma cells (Luo et al., 2018). These further reveal the miRNA regulation role in ferroptosis, which contributes to an in-depth understanding of the mechanism of ferroptosis.

## Ferroptosis in ischemic stroke

An increasing body of evidence from recently published studies suggests that ferroptosis is closely related to various diseases, such as tumor and neurological diseases (Ward et al., 2014; Fang et al., 2019; Bao et al., 2021). On the one hand, ferroptosis inducers can induce ferroptosis in abnormal cells, and tumor cells are highly sensitive to ferroptosis (Wu et al., 2019). Accordingly, ferroptosis can be induced in tumor cells to treat tumors. Non-targeted strategies based on nanoparticles have been designed to deliver iron, peroxides and other toxic substances to kill tumor cells (Zhang et al., 2019b; Raj et al., 2021).

The discovery of various enzymes regulating ferroptosis has enabled the targeted therapy of tumors, the most prominent target being GPX4, which is expressed in most tumor cells and is important for cell survival (Zhang et al., 2020b). GPX4-deficient cancer cells can be efficiently eliminated by the FSP-specific inhibitor iFSP1, while in GPX4-expressing cancer cells, iFSP1 cooperates with RSL3 to induce cancer cell ferroptosis (Gaschler et al., 2018). Therefore, FSP1 inhibitors may have clinical applications, especially for treating drug-resistant tumors or tumors that exhibit de-differentiation characteristics (Wang et al., 2021a). In addition, pharmacological or genetic inhibition of system Xc^−^ to prevent the development and metastasis of various tumors has yielded good efficacy and low toxicity in mouse models (Zhu et al., 2019).

On the other hand, ferroptosis inhibitors can inhibit ferroptosis in normal cells and can be used to prevent or treat neurological diseases (Angeli et al., 2017). Studies have found that ferroptosis is associated with *Parkinson*’s disease (Mahoney-Sanchez et al., 2021). Ferroptosis is an important cell death pathway of dopaminergic neurons (Do Van et al., 2016), and the ferroptosis inhibitor ferrostatin-1 can reportedly inhibit neuronal cell death *in vitro* and *in vivo* (Reichert et al., 2020). Interestingly, it has been reported that many pathological features of *Alzheimer*’s disease are associated with an imbalance in iron homeostasis, and iron overload in the brain may be responsible for the rapid cognitive decline in *Alzheimer*’s patients (Belaidi and Bush, 2016). Water maze experiments showed that mice with *GPX4* knockout in the cerebral cortex and hippocampal neurons showed significant cognitive impairment and degeneration of hippocampal neurons. The degree of neurodegeneration was reduced after treatment with Vitamin E or the ferroptosis inhibitor Liproxstatin-1 (Hambright et al., 2017; Lane et al., 2018). These studies suggest that ferroptosis is widely involved in regulating the functions of neurons related to learning and memory.

**FIGURE 3:**
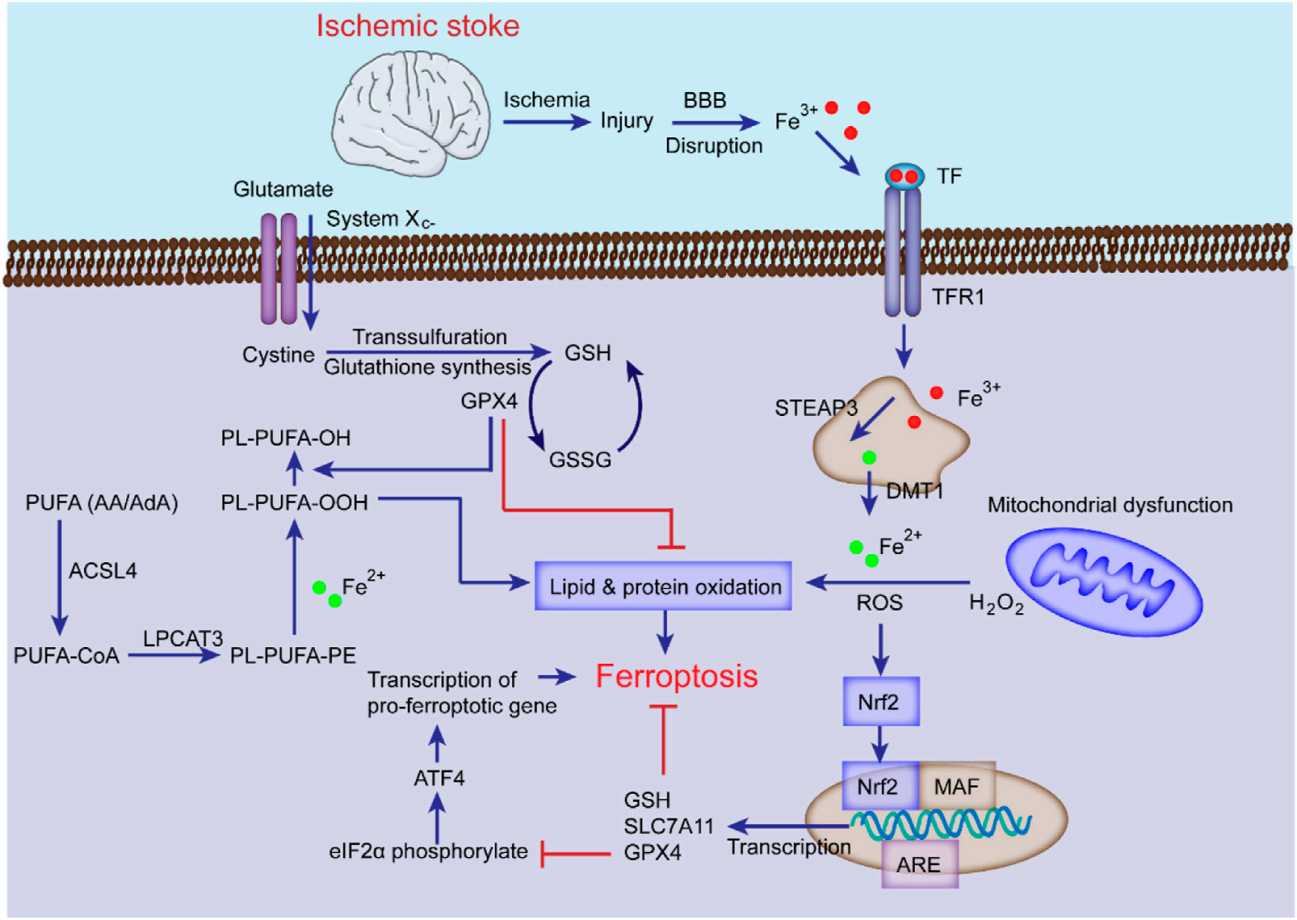
The mechanisms of ferroptosis in ischemic stroke. Following ischemic stroke, the BBB is disrupted, which allows Fe^3+^ in the blood to be released into cells through TF and TFR1, then stored in the endosome, where Fe^3+^ is converted into Fe^2+^ and transported to the cytoplasm by DMT1 with the cooperation of STEAP3. The excess Fe^2+^ generates ROS and participates in the synthesis of PUFA lipid peroxides (L-OOH), which can induce ferroptosis; System Xc^−^ is simultaneously impaired, which inhibits cystine-glutamate exchange and reduces the generation of GSH and GPX4, thereby inhibiting lipid alcohol (L-OH) production, ultimately leading to ferroptosis. Additionally, the Nrf2 pathway can inhibit ferroptosis and alleviate ischemic stroke injury by inducing GSH, SLC7A11, and GPX4 transcription.

It has been established that the levels of intracellular lipid peroxides and Fe^2+^ are increased during stroke, and ferroptosis inhibitors can upregulate the levels of GSH, GPX4 or system Xc^−^ to alleviate brain damage, indicating that ferroptosis affects the progression of stroke (Magtanong and Dixon, 2018; Zhang et al., 2021b). Studies have shown that ferroptosis inhibitors protect mice from ischemia-reperfusion injury in a middle cerebral artery occlusion (MCAO) model (Tuo et al., 2017), suggesting that ferroptosis can lead to neuronal death and NVU damage after ischemic stroke (Xu et al., 2022). Understanding the roles of iron metabolism, amino acid metabolism, and lipid metabolism of ferroptosis in ischemic stroke provides theoretical support for treating this disease (Jiang et al., 2021). The following content specifically discusses the role of ferroptosis in ischemic stroke (Figure 3).

### Iron metabolism and ischemic stroke

It is well-established that iron homeostasis in the brain is disrupted after ischemic stroke, which impedes NVU recovery (Hu and Song, 2017). Intracellular iron overload is the main mechanism for inducing ferroptosis after cerebral ischemia, and the inhibition of iron overload can suppress ferroptosis in ischemic stroke and reduce damage (Fang et al., 2018). Under pathological conditions of ischemia and hypoxia, the expression of ferritin, transferrin, and TFR1 in the brain is increased, resulting in increased iron uptake by neurons and oligodendrocytes in the NVU (Kawabata, 2019; Ng et al., 2019). The acidic environment of ischemia and hypoxia can cause the overexpression of divalent metal-ion transporter 1 (DMT1) in microglia (Rotin et al., 1986; Chang et al., 2006), resulting in increased brain iron content (Cheli et al., 2018); meanwhile, ischemia can upregulate TFR1 levels, resulting in increased iron uptake (Chen et al., 2019; Tang et al., 2021).

There is a rich literature available suggesting that serum hepcidin and iron concentrations are elevated in patients with ischemic stroke, indicating that hepcidin is critical in cerebral ischemic iron overload (Davalos et al., 1994; Petrova et al., 2016). During ischemic stroke, the expression of interleukin-6 (IL-6) in cells increases hepcidin through the JAK/STAT3 pathway (Cojocaru et al., 2009), which causes FPN1 degradation, resulting in reduced iron release and thus intensified iron accumulation in cells (Cojocaru et al., 2009; Zhou et al., 2017). Therefore, the rational application of iron metabolism inhibitors, such as deferoxamine and iron chelators, to reduce the iron content in the brain after an ischemic stroke can reduce neuronal death and promote the recovery of NVU function after ischemic stroke (Jones et al., 2020; Roemhild et al., 2021; Yang et al., 2021).

In 2017, Tuo et al. revealed the relationship between Tau and ferroptosis and the role of ferroptosis in CIRI using the MCAO mouse model (Tuo et al., 2017). Tau can promote neuronal iron efflux and inhibit ferroptosis, which may be related to the reduction of tau caused by cerebral ischemia. Meanwhile, ferroptosis inhibitors liproxstatin-1 (Lip-1) or ferrostatin-1 (Fer-1) can significantly reduce neurological damage, indicating that ferroptosis can aggravate CIRI.

After ischemic stroke, the BBB is destroyed, leading to cerebral edema and aggravating brain tissue damage and neurological dysfunction (Abdullahi et al., 2018). Numerous studies have shown that systemic iron pools are transferred to neurons in the brain parenchyma when the BBB is disrupted, thereby exacerbating ferroptosis. Accordingly, changes in iron content in brain tissue reflect the degree of BBB dysfunction (Degregorio-Rocasolano et al., 2019). Iron accumulation accompanies the entire pathological process, and iron metabolism is considered an important pathophysiological factor involved in secondary injury after ischemic stroke (Waldvogel-Abramowski et al., 2014; Roemhild et al., 2021).

### Amino acid metabolism and ischemic stroke

As the brain’s most abundant excitatory neurotransmitter, glutamate is a critical regulator in maintaining neural function (Zhou and Danbolt, 2014). NVU damage and death caused by excessively high extracellular glutamate concentration is known as excitotoxicity (Yang et al., 2019). In ischemic stroke, when the brain is in a state of ischemia and hypoxia due to metabolic disorders, glutamate release is increased, and reuptake is hindered, resulting in a rapid increase in glutamate levels in the ischemic area of the brain (Castillo et al., 1996). Subsequent activation of glutamate receptors leads to abnormal activation of several signaling pathways and iron deposition to stimulate cell death (Griesmaier and Keller, 2012; Willard and Koochekpour, 2013). Therefore, glutamate excitotoxicity is widely thought to be one of the mechanisms of ferroptosis (Zhang et al., 2021c). Indeed, glutamate excitotoxicity after cerebral ischemia, described as ferroptosis, can be effectively suppressed by the ferroptosis inhibitor Fer-1 (Xie et al., 2022).

As mentioned above, system Xc^−^ has a positive effect on inhibiting ferroptosis; however, the increase of extracellular glutamate content caused by cerebral ischemia is mainly caused by system Xc^−^ (Ikeda et al., 1989), and inhibiting the expression of system Xc^−^ can hinder ferroptosis, thereby reducing cerebral ischemia damage (Vespa et al., 1998). Domercq et al. showed that system Xc^−^ was upregulated in astrocytes and microglia in a rat model of stroke, while its inhibition reduced inflammation and attenuated CIRI (Matute et al., 2006; Martin et al., 2018). It can be concluded that the increased expression of System Xc^−^ during cerebral ischemia does not inhibit but promote the occurrence of ferroptosis, which may be due to the upregulated expression of System Xc^−^, leading to the excitotoxic effect caused by glutamate release exceeding its own antioxidant protective effect (Polewski et al., 2016).

In addition, GSH, as an endogenous inhibitor of ferroptosis, is reportedly related to ischemic stroke (Zhang et al., 2021b). Enhancing the expression of GPX4 and GSH synthesis can inhibit ferroptosis and reduce ischemic stroke injury (Zhang et al., 2021b). Increased lipid peroxidation levels and decreased GSH levels have been detected in an MCAO model (Liu et al., 2020b). Moreover, Edaravone has been proposed to counteract ferroptosis in various conditions, especially in cystine deficiency leading to decreased GSH content, and has been clinically approved for the treatment of acute ischemic stroke (Kikuchi et al., 2009; Matsumoto et al., 2018b). In addition, selenium (Se) can effectively enhance and maintain the activity of GPX4 (Ferguson et al., 2012; Liu et al., 2021). After ischemic stroke, Se supplementation can effectively inhibit GPX4-dependent ferroptosis and endoplasmic reticulum (ER) stress-induced cell death and improve NVU function by promoting GPX4 expression (Alim et al., 2019). In recent years, much emphasis has been placed on understanding the direct effects of GPX4 and GSH on stroke.

### Lipid metabolism and ischemic stroke

Lipid peroxidation is a key driver of ferroptosis, stimulated by oxidative stress (Lee et al., 2021). ROS can accumulate to toxic levels during oxidative stress, leading to cellular damage and dysfunction, whereas antioxidants can prevent cellular damage by converting ROS into harmless molecules (Conrad et al., 2018). ROS are produced in large quantities during ischemic stroke, along with decreased levels of endogenous antioxidants, leading to oxidative stress (Cadenas, 2018). For example, the 1,2,4-triazole derivative compound 11 can exert a neuroprotective effect by promoting the expression of Nrf2 and SOD to induce an antioxidant effect (Lao et al., 2022). During a stroke, AA and AdA on the cell membrane can generate lipid peroxides through a series of reactions (Mccall et al., 1987; Nishizawa et al., 2021). Growing evidence suggests that ACSL4 and LOX, especially 12/15-LOX, are increased in ischemic stroke (Jin et al., 2008; Singh and Rao, 2019).

LOX is a key enzyme that causes lipid peroxidation and induces ferroptosis (Shintoku et al., 2017). It has been reported that *12/15-Lox* gene deletion can reduce the infarct size after stroke (Singh and Rao, 2019). Additionally, 12/15-LOX were highly expressed in a cerebral ischemia model, and their inhibitors could inhibit the damage of ferroptosis to NVU cells (Jin et al., 2008). For example, the specific inhibitor ML351 has been reported to exert a protective effect against CIRI (Tourki et al., 2021). Moreover, ACSL4 participates in the synthesis and remodeling of PEs, thus affecting the synthesis of lipid peroxides, and upregulation of its expression can contribute to ferroptosis (Kuwata et al., 2019). It has been shown that in ischemic stroke, ACSL4 is upregulated and mediates neuronal death, ultimately leading to stroke injury (Li et al., 2019). Moreover, the ACSL4 inhibitor rosiglitazone can inhibit ferroptosis and protect brain function (Li et al., 2019). Cui et al. found that knockout of *ACSL4* protects mice from cerebral ischemia, whereas its overexpression can exacerbate brain damage (Cui et al., 2021). Other studies have found that the accumulation of Fe^2+^ and ROS decreased, the expression of ACSL4 and TFR decreased, and GPX4 and FTH1 increased in MCAO model cells treated with safflower flavin thus inhibiting neuronal ferroptosis in MCAO (Li et al., 2021).

These findings suggest that both ACSL4 and LOX involved in lipid metabolism can serve as innovative therapeutic targets for ischemic stroke (Cui et al., 2021), inhibiting ferroptosis by reducing ROS accumulation and lipid peroxidation, providing new ideas for the treatment of ischemic stroke (Li et al., 2021).

Additionally, mounting evidence suggests that Nrf2 is an important regulator of the cellular antioxidant defense system (Abdalkader et al., 2018), and its moderate activation is beneficial in alleviating cerebral ischemic injury (Lao et al., 2022). Although little evidence is available that changes in Nrf2 levels directly affect ferroptosis in stroke, several studies have suggested that the Nrf2 pathway can alleviate stroke injury (Shih et al., 2005; Liu et al., 2018; Lao et al., 2022). It has also been shown that Epicatechin can regulate oxidative stress through the Nrf2 pathway *via* penetrating the BBB, thus protecting against transient cerebral ischemic injury (Chang et al., 2014).

## Treatment of ischemic stroke by targeting ferroptosis

**FIGURE 4:**
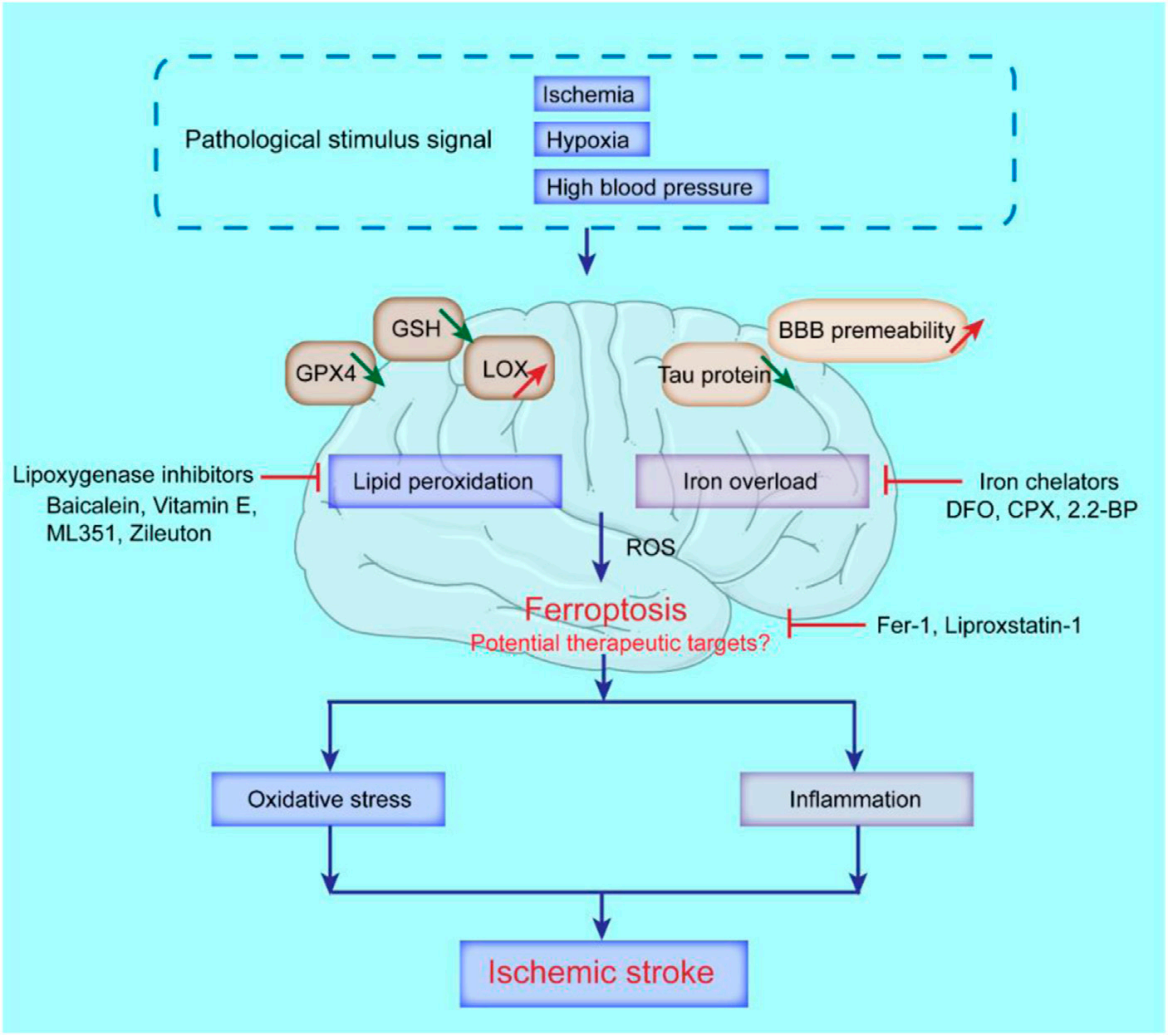
Possible molecular mechanisms of ferroptosis and potential therapeutic targets in ischemic stroke. The decrease of GSH, GPX4, tau protein, and the increase of lipoxygenase (LOX), and BBB permeability, can lead to the occurrence of ferroptosis in ischemic stroke. Iron chelators like deferoxamine (DFO), ciclopirox (CPX) and 2,2-bipyridyl (2,2-BP) can inhibit ferroptosis; Lipoxygenase inhibitors like Baicalein, Vitamin E, ML351 and Zileuton can suppress LOXs activity to rescue cells from ferroptosis; Ferrostatin-1 (Fer-1) and Liproxstatin-1 (Lip-1) inhibit radical-trapping antioxidants which activate LOXs to prevent ferroptosis in cells.

With the gradual recognition of the role of ferroptosis in ischemic stroke, treating ischemic stroke by inhibiting ferroptosis has become a research hotspot. As the pathological stimulus signals of ischemic stroke, ischemia, hypoxia and hypertension can all lead to brain damage (Khoshnam et al., 2017), during which local brain tissue metabolism changes, such as the reduction of GSH, GPX4 and tau proteins, and the increase of lipoxygenase (LOX) and BBB permeability, specifically manifested as iron overload and the enhancement of lipid peroxidation, that boost the generation of ROS and ultimately trigger ferroptosis-related cell death (Magtanong and Dixon, 2018). In addition, the restoration of normal blood circulation after ischemic stroke for a period of time will lead to CIRI (Lo and Rosenberg, 2009). Cerebral ischemia-reperfusion will lead to the activation of a variety of cell death pathways, including ferroptosis (Chen et al., 2021b). The phenomenon of increased lipid peroxidation and increased intracellular iron levels contribute to amplify the cerebral oxidative stress and inflammatory response, that further aggravate neuronal injury during reperfusion. Therefore, ferroptosis mediates and aggravates ischemic stroke injury (Figure 4). Major therapeutic advances include ferroptosis inhibitors, iron homeostasis regulators, lipid peroxidation pathway inhibitors, and ROS generation inhibitors. Table 2 summarized some key factors related to regulating ferroptosis, as well as therapeutic reagents in stroke and their functional mechanisms.

**TABLE 2:** Pharmacological research progress on ferroptosis in ischemic stroke.

Characteristics	Regulations	Reagents and mechanisms	
Morphological characteristics: Mitochondrial volume decreases, membrane density increases, and mitochondria decrease or disappear	Positive regulators: ACSL4, Hmox1, NCOA4	Inducers	Mechanism
		Erastin	Inactivates and decreases the level of GSH.
		RSL3	Inactivates GPX4 and causes accumulation of lipid hydroperoxides
		Inhibitors	
		Deferoxamine	As an iron chelator, it can prevent iron-dependent lipid peroxidation
		Liproxstatin-1	Inhibits mitochondrial lipid peroxidation and restores the expression of GSH, GPX4 and FSP1
Biochemical characteristics: Ferroptosis is manifested as GSH depletion, GXP4 inactivation, and lipid peroxide accumulation	Negative regulators: GPX4, Nrf2, HSPB1, SLC7A11, FSP1	Selenium	Protects GPX4 and upregulates GPX4 expression
		Ferrostatin-1	Prevents glutamate-induced neurotoxicity and inhibits lipid peroxidation
		Ceruloplasmin	Oxidizes ferrous ions to less toxic ferric forms
		N-Acetylcysteine (NAC)	Maintains intracellular GSH level and lower endogenous oxidant level
		Vitamin D	An antioxidant and a regulator of iron metabolism
		Vitamin E	inhibits LOX activity by competing at the substrate-binding site and by scavenging hydroxyl group radicals

Current evidence suggests that ferroptosis inhibitors Fer-1 or Lip-1 can effectively reduce brain damage after reperfusion in a mouse model of ischemic stroke (Tuo et al., 2017; Feng et al., 2019), and Edaravone can be used to treat patients with acute ischemic stroke (Enomoto et al., 2019; Kobayashi et al., 2019). Mechanistic studies have suggested that Edaravone can scavenge free radicals and inhibit lipid peroxidation, thereby inhibiting oxidative damage (Kikuchi et al., 2013). Moreover, the intravenous administration of Edaravone after I/R in rats prevented the progression of cerebral edema and cerebral infarction, alleviated the accompanying neurological symptoms, and inhibited delayed neuronal death (Watanabe et al., 2018). In recent years, iron chelation therapy has been shown to suppress ferroptosis in a CIRI model (Cappellini et al., 2006; Grignano et al., 2020). Deferoxamine (DFO), a high-affinity iron chelator with the ability to bind to free iron ions to form stable complexes that weaken the Fenton response, has been approved by the U.S. Food and Drug Administration (FDA) for the treatment of iron overload-related diseases (Hanson et al., 2009; Tevlin et al., 2021). The ferroptosis inhibitor deferoxamine, which also acts as an iron chelator, improved cognitive impairment after stroke in diabetic rats with MCAO (Hanson et al., 2009). In addition, CoQ_10_ was found to possess beneficial effects in a rat MCAO model and improved the prognosis of neurological impairment in patients with acute ischemic stroke (Nasoohi et al., 2019). Therefore, as an endogenous lipid-soluble antioxidant, CoQ_10_ can effectively inhibit lipid peroxidation and is expected to be a drug that hinders ferroptosis (Littarru and Tiano, 2007; Rizzardi et al., 2021). Another drug inhibiting ferroptosis during ischemic stroke is Se (Ramezani et al., 2021). Alim *et al.* found that Se supplementation activates GPX4 homeostatic transcription *in vivo*, inhibiting cellular ferroptosis and improving neurological function (Alim et al., 2019). Octreotide has anti-inflammatory and antioxidant effects and protects the brain from cerebral ischemic injury by activating the Nrf2/ARE pathway (Wang et al., 2015). In patients with cerebral ischemic injury, melatonin has been reported to reduce nerve cell ferroptosis by increasing Nrf2, and significantly improve the learning, memory and cognitive abilities (Koh, 2008).

Intriguingly, several traditional Chinese herbal medicines have also been shown to inhibit ferroptosis in ischemic stroke. The monoterpenoid phenol carvacrol has been reported to effectively reduce ROS expression, reduce iron deposition and elevate GPX4 levels, thereby protecting hippocampal neurons from CIRI (Friedman, 2014; Li et al., 2016). Galangin inhibition of ferroptosis by activating SLC7A11/GPX4 can reportedly attenuate CIRI (Guan et al., 2021). In addition, Naotaifang extract has been reported to inhibit neuronal ferroptosis by downregulating TFR1/DMT1 and upregulating the SCL7A11/GPX4 pathway, thereby improving neurological function in post-ischemic rats (Lan et al., 2020).

Although these traditional Chinese medicines have not been clinically validated to improve the condition of ischemic stroke patients, their safety and lack of toxicity may facilitate their clinical translation (Fung and Linn, 2015).

## Outlook

Ischemic stroke is a common disease that seriously endangers human health, and its incidence has increased in recent years (Sacco et al., 2013). Its complex pathological process and related mechanisms have become a research hotspot (Peisker et al., 2017; Yang et al., 2019). Thrombolytic therapy for cerebral ischemic injury has been limited by the narrow therapeutic time window, CIRI induction, and a high risk of hemorrhagic transformation, emphasizing the need for new treatments (Ringleb et al., 2002; Schellinger and Warach, 2004).

As the concept of NVU was proposed, researchers began to assess the feasibility of treating ischemic stroke from multiple approaches and perspectives (Cai et al., 2017; Zhao et al., 2020a). Stroke caused by different causes was regarded as a reactive injury process involving brain NVU (Yenari et al., 2010; Hu et al., 2017; Iadecola, 2017). In the treatment after brain injury, it has been transformed from single neuron protection to more comprehensive and in-depth protection of NVU (Wang et al., 2021b). The pathophysiological changes of NVU are typically characterized by tissue hypoxia, inflammation, activation of angiogenesis, and complex interactions between various components of NVU, which together lead to increased BBB permeability, brain edema, neuronal dysfunction and injury (Stanimirovic and Friedman, 2012). Cerebral ischemic injury is an inflammatory stimulus response, and all cellular components and matrix components of NVU are involved and make related responses (Ishikawa et al., 2004). The research and development of traditional drugs is limited to a certain pathological link in the pathological process of NVU, so the effect is not ideal. Therefore, during the treatment, the overall structure of NVU should be targeted, and the dynamic changes of each component should be coordinated to reduce nerve damage and promote repair. Ferroptosis is a new cell death mode discovered in recent years, with the continuous research, it has been recognized that ferroptosis plays an important role in a wide range of biological processes, including normal physiology and various pathological conditions (Stockwell et al., 2017). At present, the morphology, biology, and mechanism pathways of ferroptosis are partially understood, but the process of ferroptosis involves a variety of mechanisms, which are precisely regulated by signaling pathways. Questions remain as to how ferroptosis is related to the occurrence of diseases and whether it is associated with other modes of cell death to mediate the progression of diseases. Therefore, further in-depth study of the mechanism of ferroptosis and its role in different disease types is of great significance for finding therapeutic targets for related diseases and the development of targeted drugs.

The pathogenesis of ischemic stroke is complicated, current evidence suggests that ferroptosis plays an important role in the progression of ischemic stroke, inhibiting ferroptosis can alleviate ischemic stroke injury (Li et al., 2019; Liu et al., 2020b; Zhang et al., 2021b). When ischemic stroke occurs, iron ion aggregation in neurons leads to iron overload, which causes ROS aggregation through Fenton reaction, and GSH level is significantly reduced and lipid peroxidation increase in ischemic stroke mouse model (Liu et al., 2020b), indicating that ferroptosis is also a way of neurons death during the pathophysiological process of ischemic stroke. Ferroptosis inhibitors and iron chelators can effectively reduce the damage of neurons during ischemic brain death, suggesting that there are potential targets in the ferroptosis pathway to regulate ischemic stroke and its inhibition during ischemia has huge prospects for clinical application (Zhou et al., 2021).

However, investigating the role of ferroptosis in ischemic stroke is still in its preliminary stages, and many questions remain to be answered. For example, during ischemia and hypoxia, different cell types of NVU in brain tissue (Wang et al., 2021b), including neurons, microglia, astrocytes, oligodendrocytes, *etc.*, are stimulated and damaged (Cai et al., 2017), and ferroptosis occurs in these different cell types, although its role remains to be elucidated (Doll et al., 2017; Jiang et al., 2021; Lee et al., 2021; Nishizawa et al., 2021), warranting further studies. In the field of basic research, there is a lack of effective biomarkers for ferroptosis, such as caspase activation in apoptosis or autolysosome formation in autophagy (Earnshaw et al., 1999; Hundeshagen et al., 2011). Accordingly, exploring specific biomarkers of ferroptosis is urgent. Over the years, studies of the molecular mechanisms of ferroptosis in ischemic stroke have mostly focused on cell and animal models, indicating that more clinical studies on patients with ischemic stroke are required. In addition, drug development targeting ferroptosis in ischemic stroke is an important aspect of research. Among the ferroptosis inhibitors, only one drug, Edaraavone, is used to treat patients with acute ischemic stroke (Enomoto et al., 2019; Kobayashi et al., 2019). Other drugs have been found to be effective in animal and cell models of stroke (Hanson et al., 2009; Narayan et al., 2021). Therefore, there is an urgent need to develop and validate effective drugs in clinical treatment, including traditional Chinese medicine.

Although ferroptosis, as a new mode of cell death, plays a key role in ischemic stroke and is expected to become a new therapeutic target to improve the outcomes of this patient population, ischemic stroke is regulated by a variety of cell death pathways. Both ferroptosis and other programmed cell death modes (including cell apoptosis, necroptosis and autophagy) play an important role in the pathological process of ischemic stroke. More and more evidence has shown that there are interacting signaling pathways between these cell death modes with similar initial signals and molecular regulators. For example, p53, not only induces apoptosis, but also regulates ferroptosis (Hong et al., 2017). In the relationship between ferroptosis and necroptosis, iron overload leads to the opening of the mitochondrial permeability transition pore (MPTP), which exacerbates RIP1 phosphorylation and leads to cell necroptosis (Tian et al., 2020); HSP90 induces necroptosis and ferroptosis by promoting RIP1 phosphorylation and inhibiting GPX4 activation (Wang et al., 2018). In addition, research have shown that ferroptosis is an autophagic cell death process (Gao et al., 2016). Knockdown of autophagy-related *Atg5* and *Atg7* genes can limit Erastin-induced ferroptosis by reducing intracellular iron and lipid peroxidation, and knockdown of NCOA4 can inhibit ferritin degradation and prevent ferroptosis (Lu et al., 2017), however, the specific mechanism of autophagy mediated ferroptosis needs to be further explored. This evidence suggests a strong crosslink between them, combination therapy targeting different cell death pathways may be the most effective strategy for the treatment of ischemic stroke.
